# Angiotensin-I Converting Enzyme Inhibition and Antioxidant Activity of Papain-Hydrolyzed Camel Whey Protein and Its Hepato-Renal Protective Effects in Thioacetamide-Induced Toxicity

**DOI:** 10.3390/foods10020468

**Published:** 2021-02-20

**Authors:** Ali Osman, Abdalla El-Hadary, Aida A. Korish, Haifa M. AlNafea, Manan A. Alhakbany, Awad A. Awad, Mahmoud Abdel-Hamid

**Affiliations:** 1 Biochemistry Department, Faculty of Agriculture, Zagazig University, Zagazig 44511, Egypt; 2 Biochemistry Department, Faculty of Agriculture, Benha University, Benha 13736, Egypt; elhadary.a@fagr.bu.edu.eg; 3 Physiology Department, College of Medicine, King Saud University Medical City, King Saud University, P.O. Box 2925, Riyadh 11461, Saudi Arabia; akorish@ksu.edu.sa (A.A.K.); malhakbany@ksu.edu.sa (M.A.A.); 4 Clinical Laboratory Sciences Department, College of Applied Medical Sciences, King Saud University, Riyadh 12284, Saudi Arabia; halnafea@ksu.edu.sa; 5 Dairy Science Department, Faculty of Agriculture, Cairo University, Giza 12613, Egypt; awad.awad@agr.cu.edu.eg

**Keywords:** camel milk whey protein, papain, ACE-inhibitory activity, antioxidant activity, hepatoprotective effect, thioacetamide

## Abstract

Papain hydrolysis of camel whey protein (CWP) produced CWP hydrolysate (CWPH). Fractionation of CWPH by the size exclusion chromatography (SEC) generated fractions (i.e., SEC-F1 and SEC-F2). The angiotensin converting enzyme inhibitory activity (ACE-IA) and free radical scavenging actions were assessed for CWP, CWPH, SEC-F1, and SEC-F2. The SEC-F2 exerted the highest ACE-IA and scavenging activities, followed by CWPH. The protective effects of CWPH on thioacetamide (TAA)-induced toxicity were investigated in rats. The liver enzymes, protein profile, lipid profile, antioxidant enzyme activities, renal functions, and liver histopathological changes were assessed. Animals with TAA toxicity showed impaired hepatorenal functions, hyperlipidemia, and decreased antioxidant capacity. Treatment by CWPH counteracted the TAA-induced oxidative tissue damage as well as preserved the renal and liver functions, the antioxidative enzyme activities, and the lipid profile, compared to the untreated animals. The current findings demonstrate that the ACE-IA and antioxidative effects of CWPH and its SEC-F2 fraction are worth noting. In addition, the CWPH antioxidative properties counteracted the toxic hepatorenal dysfunctions. It is concluded that the hydrolysis of CWP generates a wide range of bioactive peptides with potent antihypertensive, antioxidant, and hepatorenal protective properties. This opens up new prospects for the therapeutic utilization of CWPH and its fractions in the treatment of oxidative stress-associated health problems, e.g., hypertension and hepatorenal failure.

## 1. Introduction

Increased blood pressure is considered the main risk factor for cardiovascular diseases. There is accumulating evidence that hypertension is strongly correlated with increased cardiac mortality [1]. Furthermore, the World Health Organization (WHO) recently reported hypertension as the leading risk factor of global mortality [2]. Angiotensin-converting enzyme (ACE) inhibitors are a famous group of antihypertensive medications that are widely used to reduce blood pressure levels. The efficacy of the antihypertensive effect of ACE inhibitors was validated [3], and they are associated with a significant reduction in major cardiovascular events and all-cause mortality [4].

Furthermore, several reports showed that certain bioactive peptides extracted from some functional food exhibit antihypertensive action due to their ACE inhibitory activity (ACE-IA) [5,6]. Camel milk proteins are considered a valuable source of multiple biologically active peptides that have diverse health benefits against several acute and chronic health problems, including the reduction of elevated blood pressure due to their antihypertensive effects [7,8,9].

Moreover, it was reported that hydrolysis of the proteins of specific food types resulted in bioactive peptides having antioxidant capacity [10]. As a point of fact, natural bioactive peptides are used in the food industry to extend the shelf life of nutritional products. This provides a safe alternative to the synthetic preservatives that carry serious health hazards, including the carcinogenic effect [11]. Alongside their importance in food manufacturing, the bioactive peptides with antioxidant actions can protect the body from the deleterious effects of the reactive oxygen species (ROS). They counteract both the peroxidation of cellular lipids and proteins [10] through scavenging of the ROS, and the stimulation of the antioxidative enzymes such as glutathione peroxidase and superoxide dismutase [12].

Bioactive peptides can be generated by various methods. Enzymatic hydrolysis is one of the commonly used methods to generate bioactive peptides from the milk proteins [13]. The release of the bioactive peptides that are encrypted in the proteins by enzymatic hydrolysis is expected to be associated with some modification in the primary structure of the protein. The latter may influence the latent activities of the resulting peptides [14,15].

In many tropical countries in Asia, Africa, and the Middle East, camels play a fundamental role in daily life. People living in the deserts use camels for transportation and as a source of milk and meat for nutrition. Besides its nutritional value, camel milk showed multiple biological and therapeutic activities [16]. Camel whey proteins (CWP) are a rich source of lactoferrin, immunoglobulins, lysozyme, α-lactalbumin, and serum albumin [17]. Several biomedical actions have been attributed to the peptides released from camel milk proteins, including antioxidant activity, ACE inhibition, and antibacterial activities against foodborne pathogenic strains [15,16,17,18]. However, the characterization of the specific bioactive peptides producing these biomedical effects and the relationship between the molecular weight of the hydrolyzed CWP fractions and their antioxidative and antihypertensive potencies have not been well investigated. Furthermore, the specific concentrations of CWP that have organoprotective potentials in the health problems associated with oxidative tissue damage need to be identified. 

Therefore, this study investigates the ACE inhibition activity (ACE-IA) and free radical scavenging actions of the bioactive peptides found in the size fractions of camel whey protein hydrolysate (CWPH) obtained by size exclusion chromatography (SEC), i.e., SEC-1 and SEC-2. Additionally, the hepatorenal protective effects of different doses of CWPH, obtained by 27% degree of hydrolysis of CWP by papain enzyme, are investigated in thioacetamide-induced toxicity in rats.

## 2. Materials and Methods

### 2.1. Preparation of CWP, CWPH, SEC-1, SCE2, and Estimation of Their Activities

#### 2.1.1. Hydrolysis and Fractionation of Camel Whey Protein (CWP)

The camel milk (Desert Research Center Farm, Matrouh City, Egypt) was subjected to centrifugation (6000× *g*, 10 min), and the resulting skimmed milk was acidified to pH 4.6 to coagulate the caseins. The CWP was separated by centrifugation and then hydrolyzed with papain (Enzyme: substrate ratio 1:200, pH 6 at 37 °C for 4 h), and the degree of hydrolysis (DH) was estimated as explained in our previous study [18]. In brief, 2,4,6-Trinitrobenzene sulfonic acid (TNBS, 0.1%, *w*/*v*) was dissolved in water; the samples and standard solutions (L-leucine at different concentrations from 0 to 2 mM) were dissolved in SDS 1%, *w*/*v*. Then, 250 mL of the tested samples or standard solutions were mixed with 2 mL phosphate buffer 0.2 M, pH 8.2 in a test tube, while 2 mL TNBS were added to each tube and incubated at 50 °C for 1h in a water bath, and 4 mL 0.1N HCl were added to each tube to stop the reaction, after which all samples were cooled to room temperature before reading the absorbance at 340 nm. The degree of hydrolysis was calculated according to Equation (1).

(1) $$\mathrm{Degree}\;\mathrm{of}\;\mathrm{hydrolysis}\;\%=\left[\;\frac{A-B}{C}\right]\times 100$$

where *A* is the amino nitrogen content in the protein before hydrolysis (mg/g protein); *B* is the amino nitrogen content in the protein after hydrolysis (mg/g protein); and *C* is the nitrogen content of the peptide bonds in the protein substrate (mg/g protein).

The camel whey protein hydrolysate (CWPH) with 27% DH was subjected to size exclusion chromatography using an FPLC system (Akta™, Uppsala, Sweden) coupled with a column (1.6 × 20 cm, Pharmacia™) filled with a Sephadex G-25 superfine grade resin for further fractionation, and the obtained fractions were referred to as SEC-1 and SEC-2. The labels CWP, CWPH, SEC-1, and SEC-2 were used in the present study.

#### 2.1.2. Estimation of the ACE Inhibition Activity (ACE-IA) 

The ACE-IA of the CWP, CWPH, SEC-1, and SEC-2 was calculated and expressed as IC_50_ (i.e., the concentration of the sample that inhibits 50% of the ACE activity) by using ABZ-Gly-Phe (NO2)-Pro as a substrate [19]. About 50 µL of the sample solution were mixed with 50 µL of the ACE solution and incubated for 10 min at 37 °C. Then, 200 µL of the substrate were mixed with the previous solution, and the mixture was kept for 30 min at 37 °C. The fluorescence was recorded at every minute of incubation using a fluorometer (Thermo Varioskan™ LUX, Vantaa, Finland) with excitation and emission wavelengths of 360 and 415 nm.

#### 2.1.3. Estimation of the Antioxidant Activity

The antioxidant activities of CWP, CWPH, SEC-1, and SEC-2 were estimated by ABTS (2,2′-azino-bis(3-ethylbenzothiazoline 6-sulfonic acid) assay (Merck KGaA, Darmstadt, Germany) and were expressed as SC_50_ (i.e., the concentration of the sample that scavenges 50% of the ABTS radicals) [20]. Five milligrams from every sample were dissolved in 1 mL deionized water and subjected to serial (2–10 folds) dilutions by ionized distilled water. The absorbance of the diluted samples was recorded at 405 nm using a Multiskan EX reader (Labsystems OY, Helsinki, Finland).

### 2.2. Estimation of the Protective Effect of CWPH on Thioacetamide-Induced Toxicity

#### 2.2.1. Animals and Experimental Design

**Figure 1:**
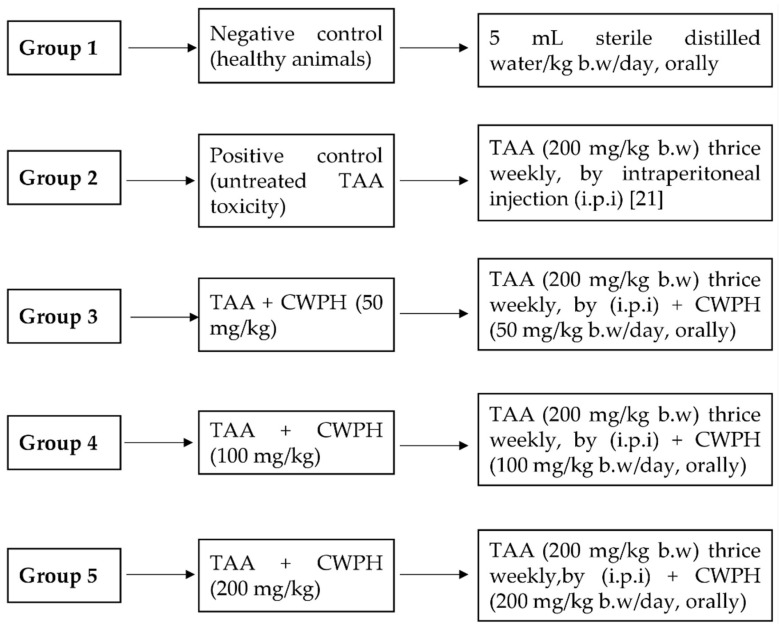
The experimental groups used for estimating the protective effects of camel whey protein hydrolysates (CWPH) on thioacetamide (TAA)-induced toxicity in rats for 8 weeks (w).

Thirty-five healthy male Wistar albino rats with body weights (BW) ranging from 120 to 140 g were obtained from the Veterinary Medicine College, Zagazig University, Zagazig, Egypt. Every 4 animals were housed in a separate cage at 25 ± 2 °C, with 12 h light/dark cycles and 50% ± 10% relative degree of humidity. The animals received a standard rodent diet (10% casein, 4% salt mixture, 1% vitamins mixture, and 85% starch) with free access to drinking water ad libitum [21]. The study design and the experimental techniques were reviewed and approved by the Institutional Review Board (IRB), namely the Institutional Animal Care and Use Committee, Zagazig University, Zagazig, Egypt (ZU-IACUC/2/F/78/2020). All animal handling and procedures followed the international guidelines of the use and care of laboratory research animals and were according to the rules of the Institutional Animal Care and Use Committee, Zagazig University. After one week of acclimatization, the animals were divided into 5 groups (*n* = 7 in each) as described in the following experimental design scheme (Figure 1).

#### 2.2.2. Blood and Tissue Sampling

At the end of the experiments (8 weeks), the animals were deprived of food overnight and were only allowed to drink water. At the time of the samples collection, the animals were anesthetized with diethyl ether inhalation, and the blood samples were collected from the retro-orbital venous plexus in plain test tubes. After blood collection, the animals underwent decapitation, and the liver was separated from all animals, which was then washed with ice-cold saline solution and sliced into two parts. One part was placed in liquid nitrogen and stored in a −80 °C deep freezer for biochemical assay of the oxidative-antioxidative parameters. The other part was preserved in a formalin solution (10% *w/v*) for histopathological examination. The collected blood was centrifuged at 3500× *g* for 10 min, and the serum samples were aliquoted and stored at −20 °C for biochemical assay.

#### 2.2.3. Biochemical Assay

##### Assessment of Liver Function, Kidney Function, and Lipid Profile

The serum levels of ALT (alanine transaminase), AST (aspartate transaminase), ALP (alkaline phosphatase), total proteins, albumin, TL (total lipids), urea, creatinine, uric acid, TG (triglycerides), TC (total cholesterol), HDL-C (high-density lipoprotein cholesterol) were assayed calorimetrically using a commercial kit purchased from Biodiagnostic Co. (Biodiagnostic Co., 29 El-Tahrer St. Dokki, Giza, Egypt) according to the manufacturer’s instructions.

The serum globulin levels were calculated according to Equation (2), as described elsewhere [21].

(2) Globulin=Total protein−Albumin

Low-density lipoprotein cholesterol (LDL-C) levels were estimated according to Equation (3):

(3) $$\mathrm{LDL}-C=\mathrm{TC}-\left[\mathrm{HDL}+\mathrm{VLDL}-C\right]$$

From this, VLDL-C (very-low-density lipoprotein) was calculated as the following Equation (4):

(4) VLDL−C=TG/5

##### Evaluation of the Redox State in the Liver

The liver was homogenized (1:9 *w*/*v*) with a 0.1 M phosphate saline buffer (pH 7.4), and the mixture was centrifuged at 4000× *g* for 15 min at 4 °C [21]. The supernatant was separated for estimation of the lipid peroxidation products malondialdehyde (MDA), glutathione peroxidase (GPx), and glutathione-S-transferase using colorimetric assay kits purchased from Biodiagnostic Co. (Biodiagnostic Co., 29 El-Tahrer St. Dokki, Giza, Egypt). The assays were performed according to the manufacturer’s instructions.

#### 2.2.4. Histopathological Examination

The preserved liver tissues were fixed in paraffin cubes, sliced into 4–5 µm slices, stained by hematoxylin and eosin (H&E) stain, and examined under light microscopy for the manifestation of TAA-induced hepatotoxicity at ×100 to ×400 magnifications.

### 2.3. Statistical Analysis

The data were expressed as mean ± SD and statistically analyzed by SPSS Software Program for Windows v. 11.0 (SPSS Ltd., Surrey, UK). Comparison between multiple groups regarding each of the studied parameters was carried out by one-way ANOVA followed by Tukey’s post hoc test. Results were considered significant at *p* < 0.05. 

## 3. Results and Discussion

### 3.1. ACE-Inhibitory Activity (ACE-IA) of CWP, CWPH, and Its (SEC-F1 and SEC-F2) Fractions

The ACE-IA of CWP, CWPH, and the two SEC-fractions (SEC-F1 and SEC-F2) are presented in Table 1 and expressed as IC_50_ (µg protein/mL). Unhydrolyzed CWP showed ACE-IA, which could be due to the presence of some bioactive peptides liberated by the bacterial contamination of the raw camel milk or by the endogenous proteolytic enzymes [22]. Similarly, Alhaj et al. [23] and Jafar et al. [24] reported ACE-IA for the untreated camel milk and the unhydrolyzed CWP. The hydrolysis of the CWP with papain significantly (*p* ˂ 0.05) enhanced its ACE-IA as a result of the augmented release of multiple bioactive peptides with ACE-IA. In agreement with our results, the hydrolysis of CWP with pepsin, trypsin, or chymotrypsin for 6 or 9 h increased its ACE-IA compared with the unhydrolyzed CWP [24]. In addition, the ACE-IA of camel milk, camel casein, and camel β-casein increased after simulated gastro-intestinal digestion or hydrolysis with papain, Alcalase, bromelain, pepsin, trypsin, or chymotrypsin [25,26].

Two fractions were obtained by fractionation of CWPH using size exclusion chromatography (SEC-F1 and SEC-F2) [18]. As shown in Table 1, SEC-F2 displayed the highest (*p* ˂ 0.05) ACE-IA with the lowest IC_50_ value. This result suggests that the smaller peptides with higher ACE-IA were concentrated in the SEC-F2. In accordance with our findings, the retentate of 3 kDa fraction of camel milk casein hydrolyzed with pepsin or chymotrypsin showed higher ACE-IA than the retentate of 5 kDa [25]. Similarly, fractions ˂ 3 kDa of camel milk fermented by *Lactobacillus rhamnosus* PTCC 1637 showed higher ACE-IA compared to the water-soluble extract of fermented camel milk [27].

### 3.2. Antioxidant Activity of CWPH and its Fractions

The antioxidant activities of CWP, CWPH, and its SEC-fractions (SEC-F1 and SEC-F2) were measured using an ABTS radical scavenging assay, and the results were expressed as SC_50_ (µg/mL) as presented in Table 1. The camel whey proteins showed antioxidant activity due to the presence of tryptophan, phenylalanine, tyrosine, histidine, and cysteine in their structure, which have the ability to scavenge the oxygen free radicals [28]. The antioxidant activity of the papain hydrolyzed CWPH was several times higher (*p* ˂ 0.05) than that of the unhydrolyzed CWP. Similarly, the hydrolysis of camel whey proteins, camel whole casein, camel β-casein, and camel αS-casein by pepsin, proteinase K, thermolysin, trypsin, or chymotrypsin increased significantly their antioxidant activities [29,30]. In addition, papain and Alcalase hydrolysates of camel casein displayed antioxidant activity [31].

The size exclusion fractions (SEC-F1 and SEC-F2) of CWPH displayed higher antioxidant activities than CWP as shown in Table 1. Interestingly, the SEC-F2, which contained the smaller peptides, exhibited the highest (*p* ˂ 0.05) antioxidant activity compared to other investigated protein forms. This could be correlated to the biological action of the amino acids incorporated into this low molecular weight fraction. These results are in line with the results of Salami et al. [25] and Addar et al. [29], who reported that the low molecular weight fractions of camel αS-casein hydrolyzed with trypsin displayed higher antioxidant activity than the high molecular weight fractions and the total hydrolysate]. Likewise, Salami et al. stated that the retentate of 5 kDa fraction of the whole camel casein hydrolyzed by pepsin, chymotrypsin, or trypsin showed higher antioxidant activity compared to the total hydrolysates [25]. Furthermore, the low molecular weight peptides of the buffalo milk protein hydrolyzed with papain, pepsin, and trypsin had higher antioxidant activity compared to unhydrolyzed proteins [20,32].

### 3.3. Effect of CWPH Treatment on Thioacetamide (TAA)-Induced Hepatorenal Toxicity

#### 3.3.1. Hepatic Oxidative-Antioxidative Balance 

The antioxidant enzymes glutathione peroxidase and glutathione S-transferase as well as the peroxidation product MDA were measured in the liver tissue homogenate of all the experimental groups. As displayed in Table 2, the G2 animals exposed to TAA 200 mg/kg without any treatment had significant (*p* < 0.05) inhibition of the antioxidant activities of the liver glutathione peroxidase and glutathione S-transferase enzymes. In contrast, the MDA levels increased significantly (*p* < 0.05) compared to negative control ( G1) animals. This could be explained in view of the reported intense oxidative stress effect of TAA intoxication that leads to the generation of excessive amounts of oxygen-free radicles [33]. In this environment, the body tries to compensate for this oxidative stress by utilizing its antioxidant enzymes. However, if the oxidative stress overwhelms the antioxidative capacity, this would lead to decreased activity of the antioxidative enzymes and a dramatic increase in the MDA levels [33]. Luckily enough, the animals treated with CWPH 50, 100 and 200 mg/kgBW exhibited significant (*p* < 0.05) improvement in the antioxidant activities of the liver glutathione peroxidase and glutathione S-transferase with decreased MDA production. This could be attributed to the strong antioxidant capacity of CWPH based on its chemical structure rich in peculiar amino acids that enhance the ROS scavenging and the antioxidative enzyme actions [20,32].

#### 3.3.2. TAA-Induced Hepatotoxicity and the Effect of CWPH Treatment

The liver is considered the main site for detoxification of the noxious chemical substances in the body. However, the metabolism of TAA by cytochrome -P450 enzyme in liver tissues produces TAA-s-oxide and TAA-S-dioxide, which induce hepatotoxicity. The latter is characterized by increased cellular permeability, inhibition of the mitochondrial activity, and swelling of the nucleoli. The consequent cellular degeneration and loss of the functional integrity of the hepatocytes result in liver failure that may progress into hepatic cirrhosis [34].

As Table 3 demonstrates, the positive control (G2) animals presented with significantly (*p* < 0.05) higher levels of the liver AST, ALT, and ALP enzymes activities, lower (*p* < 0.05) total serum proteins and globulin levels, and greater albumin/globulin (A/G) ratio as compared to the healthy negative control (G1) animals. The hepatic parenchymal injury induced by TAA resulted in oxidative damage of the hepatocytes and rupture of the mitochondria. This led to the excessive release of the liver enzymes into the circulation augmenting their activities [21,35]. The loss of the functioning hepatocytes resulted in decreased total serum proteins and globulin levels in the TAA-intoxicated (G2) animals. However, the albumin level did not decrease; rather, it tended to increase as compared to that of the control (G1) rats. This could be related to a disturbance in the metabolism of carbohydrates, proteins, and lipids or due to perturbed protein biosynthesis in the cirrhotic liver by chronic TAA-induced oxidative stress [36].

Oral administration of CWPH at three doses (50, 100, and 200 mg/kg BW/day for 8 weeks) significantly (*p* < 0.05) decreased the ALT, AST, and ALP activities in G3, G4, and G5 animals, respectively, in a dose-dependent manner compared to the untreated positive control (G2) animals. Additionally, the CWPH-treated animals showed preserved synthetic and metabolic functions of the liver resulting in the homeostasis of the serum total proteins, albumin and globulin levels, and recovery of the normal albumin/globulin ratio to the normal range. The current results indicate that CWPH at different doses (50, 100, and 200 mg/kg BW/day) counteracted the hepatoxicity of TAA. The hepatoprotective effect of CWPH could be attributed to its chemical structure rich in amino acids, vitamins, and minerals with potent antioxidant activity. Additionally, the current results indicate that the particular composition of the CWPH exerted a powerful ROS scavenging activity and boosted the activity of the antioxidative enzymes glutathione peroxidase (GPx) and glutathione S-transferase. Taken together, these mechanisms counteract the TAA-induced hepatotoxicity.

#### 3.3.3. Serum Lipid Profile

The data presented in Table 4 showed a significant (*p* < 0.05) increase in serum TL, TG, TC, LDL-C, and VLDL-C with a significant (*p* < 0.05) decrease in the serum levels of HDL-C in rats treated with TAA 200 mg/kg (G2) as compared with the negative control (G1). These results are in line with previous reports of the hyperlipidemic effect of TAA-induced toxicity [35]. The hyperlipidemic effect of TAA-toxicity could be related to the impaired fatty acid uptake and metabolism by the damaged liver cells. On the other hand, the protection of the hepatocyte function in the CWPH treated animals in G3, G4, and G5 animals resulted in significantly (*p* < 0.05) lower serum TL, TG, TC, LDL-C, and VLDL-C levels, as well as significantly (*p* < 0.05) higher serum HDL-C compared with (G2) animals. In the same manner, oral administration of camel milk protein hydrolysates exerted a similar hypolipidemic effect in streptozotocin-induced diabetic rats [37]. 

#### 3.3.4. TAA-Induced Renal Toxicity and the Effect of CWPH Treatment

As shown in Table 5 the administration of TAA to the animals in (G2) significantly (*p* < 0.05) elevated the serum urea, creatinine, and uric acid concentrations compared with the normal control (G1) animals. This demonstrated the TAA-induced renal toxicity (Table 3). However, the serum levels of urea and uric acid decreased significantly (*p* < 0.05) in G3, G4, and G5 treated with CWPH compared to the positive control (G2). The serum levels of creatinine decreased significantly (*p* < 0.05) in TAA + CWPH (100 mg/kg BW), and TAA + CWPH (200 mg/kg BW) groups compared to the positive control (G2). These results are in agreement with the findings of Abdel-Hamid et al. [38], who reported a renal protective effect of the daily oral administration of buffalo milk retentate hydrolysates at 250 and 500 mg/kg doses for 28 days in carbon tetrachloride intoxicated rats. In the present work, the renal protective action of CWPH was obtained with lower doses of 50–200 mg/kg BW and this may be related to the longer duration of the treatment in our study (8 weeks) and also to the use of the whole CWPH in comparison to the retentate used by Abdel-Hamid et al. [38].

#### 3.3.5. Histopathological Changes in the Liver

**Figure 2:**
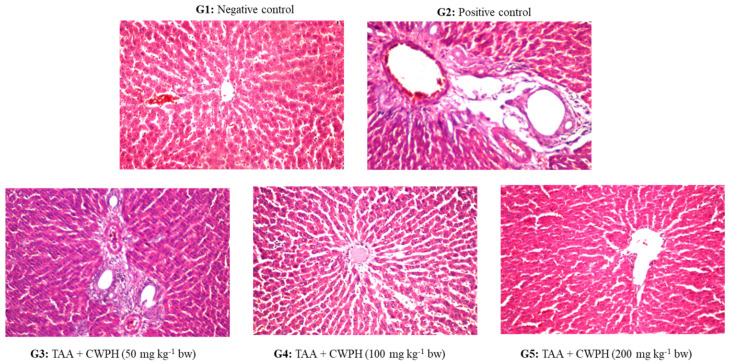
The histological picture of the liver tissue stained by hematoxylin and eosin (H&E) stain (X 400) of the healthy control and thioacetamide (TAA)-induced toxicity in rats and the effect of treatment by camel whey protein hydrolysates (CWPH) at different doses (50, 100, and 200 mg/kg BW) for 8 weeks. G1: Normal control; G2: Positive control (TAA at 200 mg/kg BW); G3: TAA + CWPH (50 mg/kg BW); G4: TAA + CWPH (100 mg/kg BW); G5: TAA + CWPH (200 mg/kg BW).

Figure 2 presents the histological pictures of the liver sections of the studied groups at the end of the experiment. The liver section of the negative control (G1) showed the normal histological appearance of the central vein and the surrounding hepatocytes in the liver parenchyma. However, the liver sections of the positive control (G2) rats with untreated TAA toxicity demonstrated moderate hepatocellular degenerative changes, mainly diffuse centrilobular necrosis around the central vein all over the hepatic parenchyma. The portal area showed dilatation in the portal vein as well as dilatation in the bile ducts with fibrosis. Treatment by CWPH at 50 mg/kg BW was not effective in improving the histopathological picture of the TAA-induced hepatotoxicity in (G3) animals, which showed fibrosis in the portal area. However, the animals in (G4) and (G5) receiving TAA + CWPH (100 and 200 mg/kg BW, respectively) showed marked improvement in the histopathological picture of the liver in association with the biochemical improvement in their liver function. The amelioration of the histopathological picture of TAA-induced hepatotoxicity by the long-term administration of high doses of CWPH could be related to the potent antioxidant properties and ROS scavenging activities of the bioactive peptides in CWPH that halt the progression of the oxidative stress-associated damage of TAA on the hepatocytes.

## 4. Conclusions

The CWPH and its smaller fractions showed potent ACE-IA and antioxidant properties. Interestingly, the small molecular weight fraction (SEC-F2) of CWPH demonstrated the highest ACE-IA and free radical scavenging actions in comparison to the bigger fractions. The administration of CWPH ameliorated the oxidative hepatorenal damage associated with TAA toxicity. The results of the current study demonstrated a considerable therapeutic potential for CWP and its hydrolysate derivatives as antihypertensive and hepatorenal protective agents.

## Figures and Tables

**Table 1:** ACE-inhibition and free radical scavenging activities of camel whey protein (CWP), camel whey protein hydrolysates (CWPH), and its fractions.

Samples	ACE-IA (IC_50_; µg/mL)	Radical Scavenging (SC_50_; µg/mL)
CWP	576.7 ± 6.5 ^a^	489 ± 3.6 ^a^
CWPH	410.8 ± 2.7 ^c^	79.8 ± 1.9 ^c^
SEC-F1	469.3 ± 4 ^b^	317.6 ± 2.5 ^b^
SEC-F2	179.9 ± 2.6 ^d^	60.9 ± 2.9 ^d^

Values are means ± SD of three experiments. Values in the same column with different superscript letters are significantly different from each other (*p* < 0.05). SEC-F1 and SEC-F2 are size exclusion chromatography numbers 1 and 2.

**Table 2:** The oxidative–antioxidant balance in normal and thioacetamide (TAA) intoxicated rats and the effect of 8 weeks treatment by camel whey protein hydrolysate (CWPH).

Groups	MDA (mol/mg)	Glutathione Peroxidase (U/g Tissue)	Glutathione S-Transferase (U/g Tissue)
G1	3.55 ± 0.39 ^c^	2.64 ± 0.06 ^b^	3.50 ± 0.10 ^ab^
G2	10.21 ± 0.39 ^a^	0.98 ± 0.06 ^e^	2.55 ± 0.10 ^c^
G3	5.94 ± 0.39 ^b^	2.12 ± 0.06 ^d^	3.23 ± 0.10 ^b^
G4	4.15 ± 0.39 ^c^	2.37 ± 0.06 ^c^	3.56 ± 0.10 ^a^
G5	3.88 ± 0.39 ^c^	2.93 ± 0.06 ^a^	3.74 ± 0.10 ^a^

Values in the same column with different superscript letters are significantly different (*p* < 0.05). G1: Normal control; G2: Positive control (TAA at 200 mg/kg BW); G3: TAA + CWPH (50 mg/kg BW); G4: TAA + CWPH (100 mg/kg BW); G5: TAA + CWPH (200 mg/kg BW).

**Table 3:** The changes in serum ALT, AST, ALP, and protein profile in thioacetamide (TAA)-induced toxicity in rats after 8 weeks of treatment by camel whey protein hydrolysate (CWPH).

Groups	ALT Activity (U/L)	AST Activity (U/L)	ALP (U/L)	Total Proteins (g/dL)	Albumin (g/dL)	Globulin (g/dL)	A/G Ratio
G1	45.00 ± 4.41 ^bc^	23.33 ± 4.89 ^c^	81.86 ± 14.30 ^b^	7.74 ± 0.31 ^a^	2.89 ± 0.34 ^ab^	4.85 ± 0.28 ^c^	0.60 ± 0.13 ^b^
G2	160.00 ± 4.41 ^a^	151.67 ± 4.89 ^a^	335.86 ± 14.30 ^a^	5.35 ± 0.31 ^b^	3.94 ± 0.34 ^a^	1.41 ± 0.28 ^d^	2.86 ± 0.13 ^a^
G3	55.00 ± 4.41 ^b^	45.00 ± 4.89 ^b^	109.54 ± 14.30 ^b^	8.65 ± 0.31 ^a^	2.60 ± 0.34 ^b^	6.05 ± 0.28 ^ab^	0.43 ± 0.13 ^b^
G4	33.33 ± 4.41 ^c^	36.67 ± 4.89 ^bc^	94.38 ± 14.30 ^b^	8.48 ± 0.31 ^a^	2.89 ± 0.34 ^ab^	5.59 ± 0.28 ^bc^	0.53 ± 0.13 ^b^
G5	28.33 ± 4.41 ^d^	31.67 ± 4.89 ^bc^	74.72 ± 14.30 ^b^	8.75 ± 0.31 ^a^	1.92 ± 0.34 ^b^	6.83 ± 0.28 ^a^	0.30 ± 0.13 ^b^

Values in the same column with different superscript letters are significantly different from each other (*p* < 0.05). G1: Normal control; G2: Positive control (TAA at 200 mg/kg BW); G3: TAA + CWPH (50 mg/kg BW); G4: TAA + CWPH (100 mg/kg BW); G5: TAA + CWPH (200 mg/kg BW). A/G (albumin/Globulin).

**Table 4:** Serum lipid profile in normal and thioacetamide (TAA)-intoxicated rats and the effect of treatment by camel whey protein hydrolysate (CWPH) for 8 weeks.

Groups	Total Lipid (mg/dl)	Triglyceride (mg/dl)	Total Cholesterol (mg/dl)	HDL-C (mg/dl)	LDL-C (mg/dl)	VLDL-C (mg/dl)
G1	491.83 ± 12.54 ^b^	151.56 ± 7.65 ^b^	96.78 ± 1.87 ^c^	39.38 ± 2.24 ^b^	27.09 ± 1.27 ^c^	30.31 ± 1.53 ^b^
G2	663.25 ± 12.54 ^a^	274.83 ± 7.65 ^a^	147.03 ± 1.87 ^a^	27.03 ± 2.24 ^c^	65.03 ± 1.27 ^a^	54.97 ± 1.53 ^a^
G3	493.19 ± 12.54 ^b^	160.20 ± 7.65 ^b^	111.89 ± 1.87 ^b^	35.06 ± 2.24 ^b^	44.79 ± 1.27 ^b^	32.04 ± 1.53 ^b^
G4	503.77 ± 12.54 ^b^	147.88 ± 7.65 ^b^	107.63 ± 1.87 ^bc^	37.12 ± 2.24 ^b^	40.93 ± 1.27 ^b^	29.58 ± 1.53 ^b^
G5	464.76 ± 12.54 ^b^	143.26 ± 7.65 ^b^	100.42 ± 1.87 ^c^	50.13 ± 2.24 ^a^	21.64 ± 1.27 ^c^	28.65 ± 1.53 ^b^

Values in the same column with different superscript letters are significantly different (*p* < 0.05). G1: Normal control; G2: Positive control (TAA at 200 mg/kg BW); G3: TAA + CWPH (50 mg/kg BW); G4: TAA + CWPH (100 mg/kg BW); G5: TAA + CWPH (200 mg/kg BW).

**Table 5:** The renal function biomarkers in normal and thioacetamide (TAA)-intoxicated rats and the effect of 8 weeks treatment by camel whey protein hydrolysate (CWPH).

Groups	Uric Acid (mg/dL)	Urea (mg/dL)	Creatinine (mg/dL)
G1	5.70 ± 0.58 ^b^	39.93 ± 4.72 ^c^	1.08 ± 0.16 ^c^
G2	9.09 ± 0.58 ^a^	68.52 ± 4.72 ^a^	3.25 ± 0.16 ^a^
G3	7.47 ± 0.58 ^b^	59.06 ± 4.72 ^ab^	2.20 ± 0.16 ^b^
G4	6.74 ± 0.58 ^b^	48.36 ± 4.72 ^bc^	1.45 ± 0.16 ^c^
G5	6.19 ± 0.58 ^b^	44.41 ± 4.72 ^bc^	1.19 ± 0.16 ^c^

Values in the same column with different superscript letters are significantly different (*p* < 0.05). G1: Normal control; G2: Positive control (TAA at 200 mg/kg BW); G3: TAA + CWPH (50 mg/kg BW); G4: TAA + CWPH (100 mg/kg BW); G5: TAA + CWPH (200 mg/kg BW).

## Data Availability

Data available upon request.
